# Spectral CT of the abdomen: Where are we now?

**DOI:** 10.1186/s13244-021-01082-7

**Published:** 2021-09-27

**Authors:** Sharon Z. Adam, Aviad Rabinowich, Rivka Kessner, Arye Blachar

**Affiliations:** 1 Department of Diagnostic Radiology, Tel Aviv Sourasky Medical Center, 6 Weizmann St., 6423906 Tel Aviv, Israel; 2 Sackler School of Medicine, Tel Aviv University, Tel Aviv, Israel

**Keywords:** Spectral CT, Dual energy CT, Abdominal Imaging, Mono-energetic images, Iodine maps

## Abstract

Spectral CT adds a new dimension to radiological evaluation, beyond assessment of anatomical abnormalities. Spectral data allows for detection of specific materials, improves image quality while at the same time reducing radiation doses and contrast media doses, and decreases the need for follow up evaluation of indeterminate lesions. We review the different acquisition techniques of spectral images, mainly dual-source, rapid kV switching and dual-layer detector, and discuss the main spectral results available. We also discuss the use of spectral imaging in abdominal pathologies, emphasizing the strengths and pitfalls of the technique and its main applications in general and in specific organs.

## Key points

- Spectral CT allows for material-specific analysis in addition to anatomical images of conventional CT.
- The main spectral acquisition techniques are dual source, rapid kV switching and dual layer detector.
- The most commonly used spectral applications are virtual non-contrast images, mono-energetic images at different keV levels and iodine concentration.
- Spectral data can be used to accentuate enhancement, reduce metal artifacts, decrease the number of scans in multiphasic imaging and characterize lesions.

## Introduction

Spectral computed tomography (CT), also known as multi-energy CT or dual-energy CT, was first hypothesized in the 1970s. However, it took years until the first multi-energy scanners were robust enough to be introduced into routine clinical practice in 2006, first as dual source scanners (utilizing two tubes with different voltages) and rapid kilo-voltage (kV) switching scanners, and lately also as dual layer detector scanners.

Conventional CT provides anatomical and morphological evaluation of organs and tissues, with mostly qualitative assessment of tissues in relation to adjacent tissues, considering that the images are dependent on the energy level selected during acquisition. Spectral CT adds a new dimension to the images acquired, with material decomposition supplying data regarding true composition of specific materials in the scanned tissues, allowing for accurate assessment of iodine concentration, subtraction of iodine from contrast-enhanced images, and also supplying better quality anatomical images using mono-energetic reconstructions.

We will discuss the principles and technical aspects of spectral CT acquisition, describe general advantages and disadvantages and also refer to organ specific applications in the abdomen.

## Spectral principles and technology

Conventional CT images are generated from average attenuation values in multiple voxels which contain multiple tissues. These tissues may have overlapping attenuation at any given peak tube voltage (kVp). The main contributors to attenuation coefficients are the photoelectric effect, which predominates in lower energy levels, is highly energy-dependent and related to high atomic numbers, and Compton scattering, which occurs at energies above 50 kilo-electron Volt (keV), and is predominantly related to the electron density of a given material. The comparison of attenuation levels derived from two energy levels, high and low, can be utilized to calculate the photoelectric effect and Compton scattering. This allows for separation of tissues with similar attenuation in any single energy level. This is commonly referred to as material decomposition and is the basis for spectral CT imaging.

**Fig. 1:**
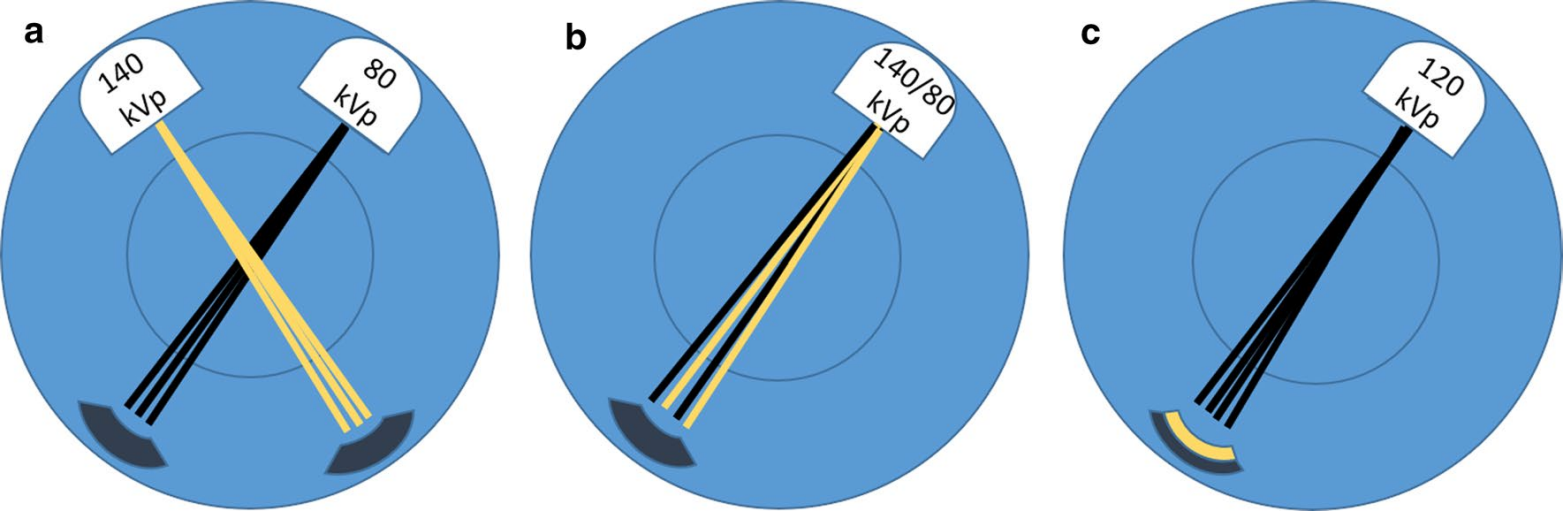
The main acquisition techniques currently in the market for spectral imaging: dual source dual energy CT (**a**), rapid kV switching (**b**) and detector based spectral imaging (**c**)

The datasets of the two energy levels can be acquired in multiple ways. The three main acquisition techniques currently in the market (Fig. 1) are—dual source acquisition (in which a low kV tube and a high kV tube scan simultaneously at an angle offset to achieve 2 energy spectra), rapid kV switching (in which the same tube rapidly switches from low to high kV constantly during its rotation) and detector-based spectral separation (in which the detector is a dual layer detector separating the energy levels after the beam has crossed the patient, detecting low energy photons in the top layer and high energy photons in the bottom layer), which is the newest technique, available commercially since 2016. Other techniques also exist, although not as commonly used, including "rotate-rotate" techniques (in which the tube alternates energy levels between rotations) and scanning using filter-based spectral beam splitting (split gold-tin filter resulting in spectral separation before the beam passes through the patient), which has also only recently been introduced into clinical practice [1].

Each technique and each manufacturer uses a different mathematical calculation to achieve spectral separation, thus resulting in different spectral separation accuracies [2], but all are considered robust and are in use clinically.

There are several main advantages and disadvantages to the different techniques. Detector-based spectral imaging has the advantage of acquiring spectral data for all scans without predetermining the need for spectral data ("always on"), with spectral data being able to be acquired retroactively, without changing scan protocols and available for all scan phases in multiphase studies. These scanners also provide true conventional CT images, unlike all other techniques which only provide the conventional-equivalent image which is a blended images of the two energy levels. Detector-based imaging also has the best temporal coherence because the different energies are all detected at the same time, and is affected the least by patient motion. Dual source spectral imaging has the advantage of two tubes, able to generate good image quality even in large patients, with the second tube able to be used in non-spectral mode to perform extremely fast scans, while also being the only technique limited by a small field-of-view for spectral data because of space limitations within the gantry, approximately 35 cm compared with 50 cm for other techniques. The main spectral acquisition techniques are able to provide scans with comparable radiation doses to single-energy CT, regardless of the technique [3–5]. A full comparison of technologies with their advantages and disadvantages is beyond the scope of this article and can be found in the literature [1, 6].

## General applications

The main applications currently available for most spectral scanners are virtual non-contrast (VNC) images, mono-energetic images at different keV levels, iodine concentration, effective atomic number images, and other material density images, which are constantly being improved, with new applications also being developed. Some applications will be discussed separately here, while others will be discussed in the organ-specific section later in the article.

### VNC images

VNC images are created by subtracting all contrast-enhanced structures from the acquired image. A robust VNC image will allow for reduction of the overall radiation exposure by decreasing the number of scans per protocol. This reduction can be substantial, ranging from 25 to 35% [7], depending on site-specific protocols. VNC images are not affected by concentration or rate of injected contrast or by the phase they are acquired from (arterial vs. portovenous phase) [8].

**Fig. 2:**
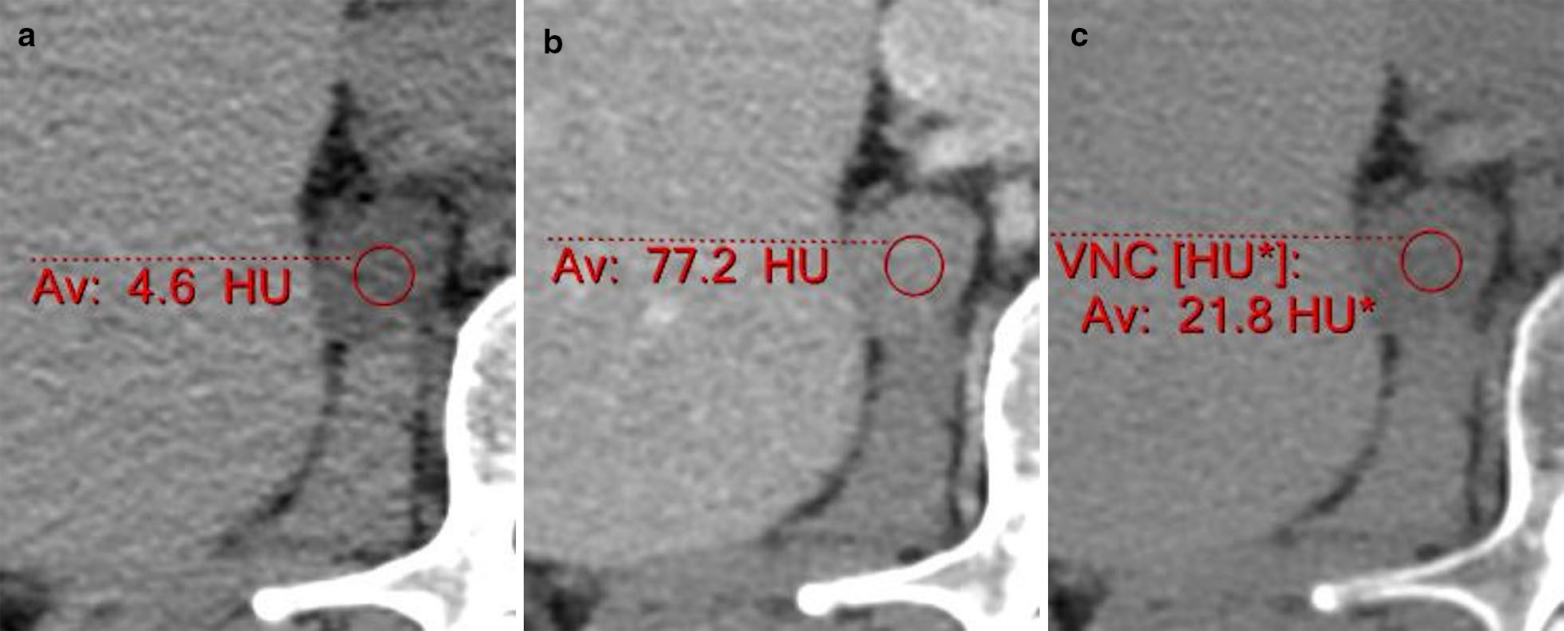
Adrenal CT of a 52-year-old woman showing a 21 mm nodule in the right adrenal gland. The nodule has an attenuation of 4.6 HU on TNC images (**a**), 77.2 HU on portovenous phase images (**b**) and 27 HU on 15 min delayed images (not shown), proving this is adenoma both by showing an absolute washout of 69% and by pre-contrast attenuation. However, VNC images show attenuation of 21.8 HU (**c**), making this an indeterminate nodule had this been an incidentaloma on portovenous phase spectral imaging only

However, replacing true unenhanced (true non-contrast, TNC) images by VNC images is only possible if we can use them in a similar fashion. Studies have shown that VNC images have comparable and adequate image quality [7, 9, 10]. It has also been shown that there is good correlation between attenuation on TNC images and VNC images [7, 9–11]. However, when one looks at the differences in attenuation numbers, quoted as under 15 HU in 92.6–98.6% of measurements, with higher attenuation differences in fluid and fat [7, 9], one wonders as to the clinical applicability. TNC images in multiphasic examinations are used mainly for qualitative analysis as for the presence of calcifications and other hyperdense substances prior to contrast administration, assessment of pre-contrast attenuation in adrenal lesions or liver parenchyma, and quantitative analysis as part of enhancement measurements in a suspected lesion. As for evaluation of enhancement, one can argue that the inability to use the VNC as baseline for enhancement measurement is off-set by the data provided for iodine concentration. However, this is not the case for evaluation of calcifications, for instance in evaluation of arteries for calcified plaques and determination of severity of vessel occlusion. It is even more concerning in adrenal incidentalomas (Fig. 2), one of the most common lesions encountered in clinical practice. A possible difference of 15 HU, even when taking into account the positive bias in fat containing tissues (consistently higher attenuation in VNC compared with TNC) [9], may cause over diagnosis of adenomas as uncertain lesions and lead to unnecessary further imaging [10]. A more substantial body of knowledge is needed as for the correct threshold levels in such cases before the substitution can be made.

Therefore, it appears that VNC images are still not robust enough to replace TNC images in most clinical settings. We suggest such a substitution only in cases of borderline additional information gained by adding an unenhanced image. However, VNC images are a valuable tool in monophasic post-contrast protocols, in which previously many incidentalomas led to further imaging, which now can be avoided in some cases.

### Mono-energetic images

The mono-energy application simulates a scan using a monochromatic x-ray beam and allows us to choose the energy level, in keV, for the reconstruction. Available energy levels vary between spectral acquisition method and manufacturer, ranging between 40 and 200 keV. The energy level equivalent to 120 kVp is approximately 75 keV.

Conventional-equivalent mono-energetic images have been shown to have improved signal-to-noise ratio (SNR) and contrast-to-noise ratio (CNR) in contrast-enhanced scans, particularly in obese patients [12–14]. However, one must take into account the fact that measurements of HU cannot be used as with conventional images, and evaluating enhancement needs to be performed using iodine concentration images.

The attenuation of structures increases as the mean energy of the photons decreases, which is true for both non-spectral low kVp scanning and dual energy low keV mono-energetic settings. This is especially important for scans using contrast media, as a decrease in energy in the range approaching the K-edge of iodine (which is 33 keV) causes markedly increased attenuation of iodine-enhanced structures [8].

Thus in spectral imaging, low keV mono-energetic images accentuate iodine-enhanced structures without increasing image noise and this allows us to decrease the amount of injected contrast media, which may be crucial in patients with renal impairment [8, 15–23]. Studies have shown a feasible reduction of 70% in vascular studies and 50% in non-vascular studies, while considering that for optimal evaluation of hypervascular lesions the reduction should likely be more moderate, of approximately 35% [8]. However, the decision to decrease contrast doses is more complex, and needs to take into account overall injected volume and flow rate needed to achieve optimal enhancement. Also, as many institutions use multiple scanners only some of which are spectral scanners, this may entail individualized contrast protocols per scanner and per pathology (not only per body part), which may prove to be problematic for optimal workflow and may lead to more errors. Also one must consider that as the attenuation of injected contrast increases, the attenuation of positive oral contrast also increases [24, 25] which may cause more artifacts.

**Fig. 3:**
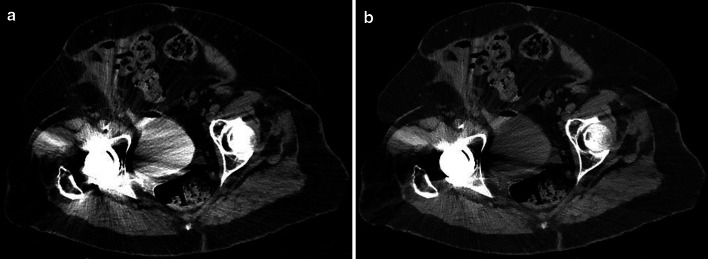
CT cystography of a 70-year-old woman after renal transplantation with a prior total hip replacement. Beam hardening artifacts caused by metal hardware markedly degrade evaluation of pelvic structures (**a**). Spectral mono-energetic image at 140 keV shows improvement of artifacts, at a price of reduced attenuation of contrast in the bladder (**b**)

At the other end of the spectrum, high energy levels decrease beam-hardening artifacts and can be used for metal artifact reduction (Fig. 3) [26–28]. For both low keV and high keV reconstructions, one can choose the optimal energy level based on personal preference, and at our institution, using dual-layer spectral imaging, we prefer 50 keV and 140 keV images.

### Iodine concentration images (iodine maps)

**Fig. 4:**
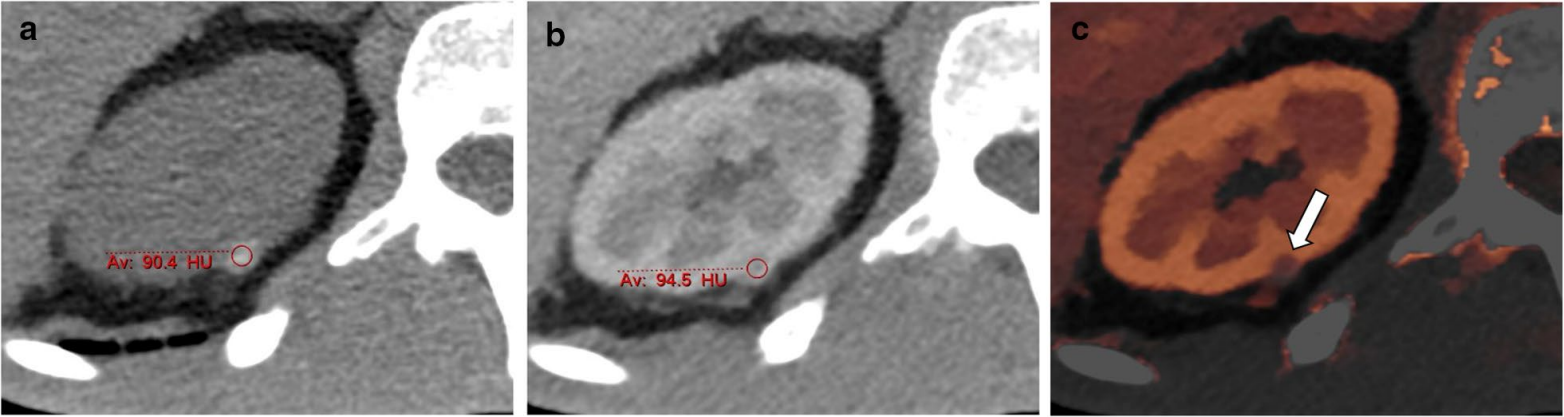
An incidentally identified hyperdense right renal cyst in a 72-year-old man. Axial TNC image shows a markedly hyperdense cyst, most consistent with a hemorrhagic cyst (**a**) without enhancement on the portovenous phase image (**b**). However, iodine overlay maps show suspected mild enhancement (**c**). The iodine content of the cyst was 1.3 mg/mL, which by accepted thresholds would be considered an enhancing lesion, however, the normalized iodine content was only 0.27 mg/mL, consistent with lack of enhancement seen on conventional images

This is a very significant application for lesion characterization. The generated images are equivalent to MRI subtraction images. Iodine maps give radiologists the confidence in evaluating contrast enhancement of a lesion, proving that some lesions do not show contrast material uptake and are benign, thus obviating the need for further evaluation. These images have been shown to have high accuracy regardless of acquisition parameters, as long as the radiation dose is within a clinical range [29, 30]. Although differences in accuracy between manufacturers (likely related to the difference in acquisition technique) have been reported for phantoms with errors in measurement questioning our ability to use reported threshold levels for certain pathologies [2, 31, 32], the error is under 10%, which is an acceptable error, as long as correct thresholds are used. The correct threshold has not been adequately established yet. One recent study found liver cysts to have iodine uptake of 0.23 ± 0.31 mg/mL, consistent with the accepted threshold of 0.5 mg/mL across all scanners, but also found differences of up to 20% within the same lesion in different time points [33]. Another recent study [34] has found higher thresholds of 1.3 mg/mL, and has suggested a more accurate approach of measuring iodine concentration by normalizing the results to the iodine concentration of the aorta, and using a normalized threshold of 0.3 mg/mL. Until further data is accumulated, we believe radiologists should choose between a more conservative approach, using a lower threshold for enhancement and likely suggesting further imaging for lesions showing suspected mild iodine uptake, and a more liberal approach using a higher threshold which may lead to lesions being incorrectly diagnosed as non-enhancing. At our institution, where we use detector-based spectral CT, we choose the conservative approach. We also believe iodine concentration measurement on contrast-enhanced only studies, are not robust enough for omitting the unenhanced phase of multiphasic protocols (Fig. 4).

One of the most important uses for iodine maps in abdominal imaging is characterization of incidentally identified small lesions, the so called "too small to characterize" lesions. Although it is tempting to say iodine maps can be used to determine whether these small lesion are truly non-enhancing, one must remember that these images are still affected by the same partial volume artifacts which prevent us from reliably measuring the density (HU) of a lesion on conventional images. The true size threshold for determining the iodine content of a lesion using iodine maps is unknown.

## Organ specific applications

### Liver

**Fig. 5:**
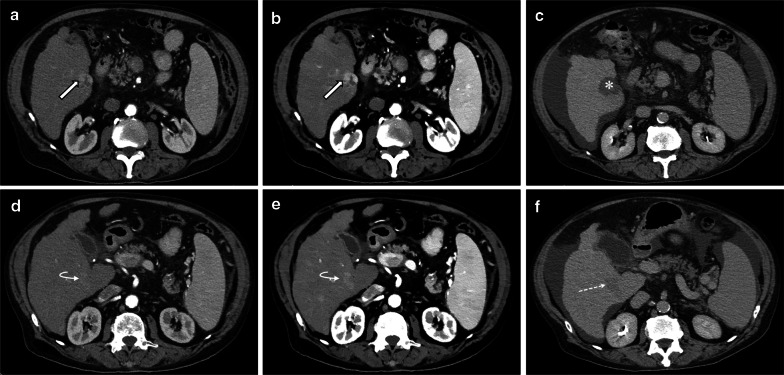
Multiphasic spectral CT of a 73-year-old man with known HCC. The known tumor (arrows) shows typical hypervascularity on the conventional arterial phase (**a**) which is more prominent on 50 keV mono-energetic image (**b**), and was ablated with good treatment response (asterisk) on the 1-month follow-up (**c**). However, there was an additional lesion (curved arrow) which should have been suspected by the radiologist on baseline imaging but was missed owing to its minimal arterial enhancement (**d**). This lesion would not have been missed if 50 keV mono-energetic images had been reviewed prospectively (**e**), and on follow-up imaging showed definite washout and enlargement (dashed arrow), when the patient returned with advancing disease including tumor thrombus in the portal vein

The most intuitive use of spectral CT in the liver is for better visualization of hypervascular lesions using low keV mono-energetic images of the arterial phase (Fig. 5). This applies to hypervascular tumors, such as hepatocellular carcinoma (HCC) and neuroendocrine tumors (NET), with studies showing better conspicuity, allowing for visualization of a larger number of lesions, with improved reader confidence [35–38]. However, the low keV images may reveal many small hypervascular lesions, some of which may be of uncertain significance, and therefore evaluation of such lesions should always be in the correct context, with concurrent evaluation of conventional images, in order to prevent over diagnosis. Using low keV images may also increase visualization of hypo-enhancing hepatic lesions, such as small hypovascular metastases, by increasing the attenuation of the surrounding liver [39].

Better visualization of blood vessels and extravasation on low keV images [17, 40] may lead to improved visualization of active hepatic hemorrhage in both traumatic and non-traumatic cases and may also lead to better evaluation of vasculature before and after liver transplantations.

Although iodine maps have been shown to be accurate in liver phantom models, their clinical use in evaluation of tumor response is still limited because of differences in the biological distribution causing inter-patient and intra-patient variability [33]. However, iodine quantification has been shown to improve accuracy of diagnosis of portal vein tumor thrombus, best evaluated using a threshold level of 0.9 mg/mL [41]. It may also potentially help to evaluate response to treatment in Budd-Chiari syndrome [42] and differentiate between liver metastases and abscesses [43]. Specifically for HCC, it has been shown that iodine concentration images improve the assessment of hypervascularity and washout [44]. It is also showing promise in differentiating between HCC and non-cancerous hypervascular cirrhotic nodules based on iodine content alone, regardless of washout, using a threshold of 1.99 for iodine concentration normalized to the adjacent liver [45], although this still needs further verification before implementation in clinical practice. Iodine concentration in the equilibrium phase has been proposed as a method for the noninvasive evaluation of fibrosis based on the expansion of the interstitial space seen in cirrhosis [46].

Hepatic steatosis can also be diagnosed using material decomposition, and fat can be quantified accurately using this method [47–52].

### Pancreas

**Fig. 6:**
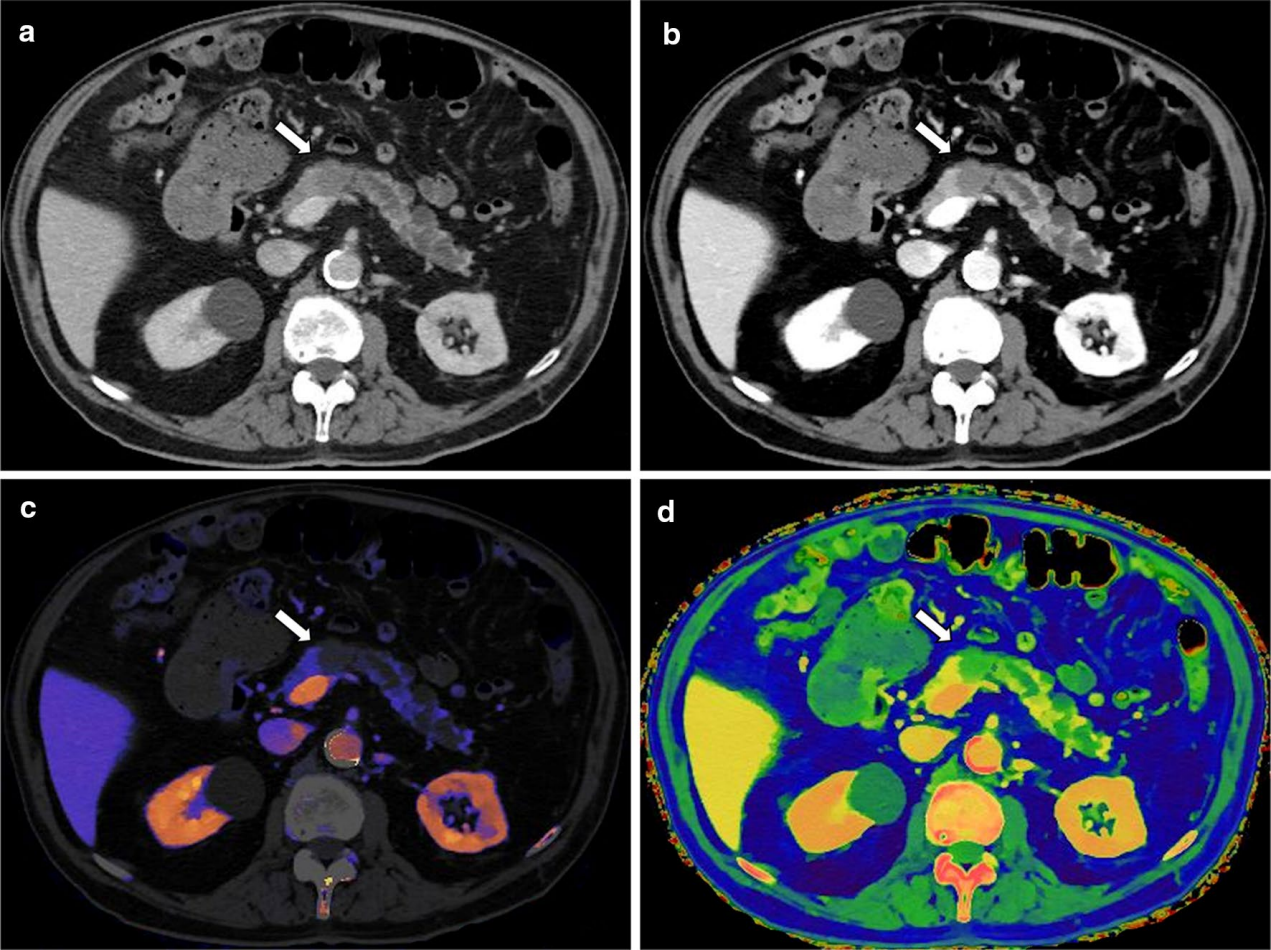
Pancreatic adenocarcinoma in an 84-year-old man. On the late arterial phase image there is a lesion in the pancreatic body showing mild hypoenhancement compared to the normal pancreatic tissue (**a**). The lesion's conspicuity is markedly improved using 50 keV mono-energetic images (**b**), iodine concentration overlay maps (**c**), and effective atomic number maps (**d**)

Pancreatic adenocarcinoma is often difficult to visualize on conventional CT images, with only a vague hypodensity visible even on pancreatic phase images. Low keV spectral images and iodine quantification have been shown to improve the detection of pancreatic adenocarcinoma (Fig. 6) and also improve evaluation of arterial involvement [53–56].

Pancreatitis has also been studied, and in acute pancreatitis iodine quantification may aid in diagnosis, using a threshold of 2.1 mg/mL on pancreatic phase images [57] and also in assessing vascular complications and necrosis, using low keV images [58]. Differentiating chronic mass forming pancreatitis from adenocarcinoma is more problematic, but iodine quantification also shows promise in this regard [59]. In patients with chronic pancreatitis VNC images may aid in detecting pancreatic calcifications and ductal calculi on single phase contrast enhanced scans, although theoretically these may underestimate small calcifications because of erroneous calcium subtraction [58, 60].

### Biliary system

**Fig. 7:**
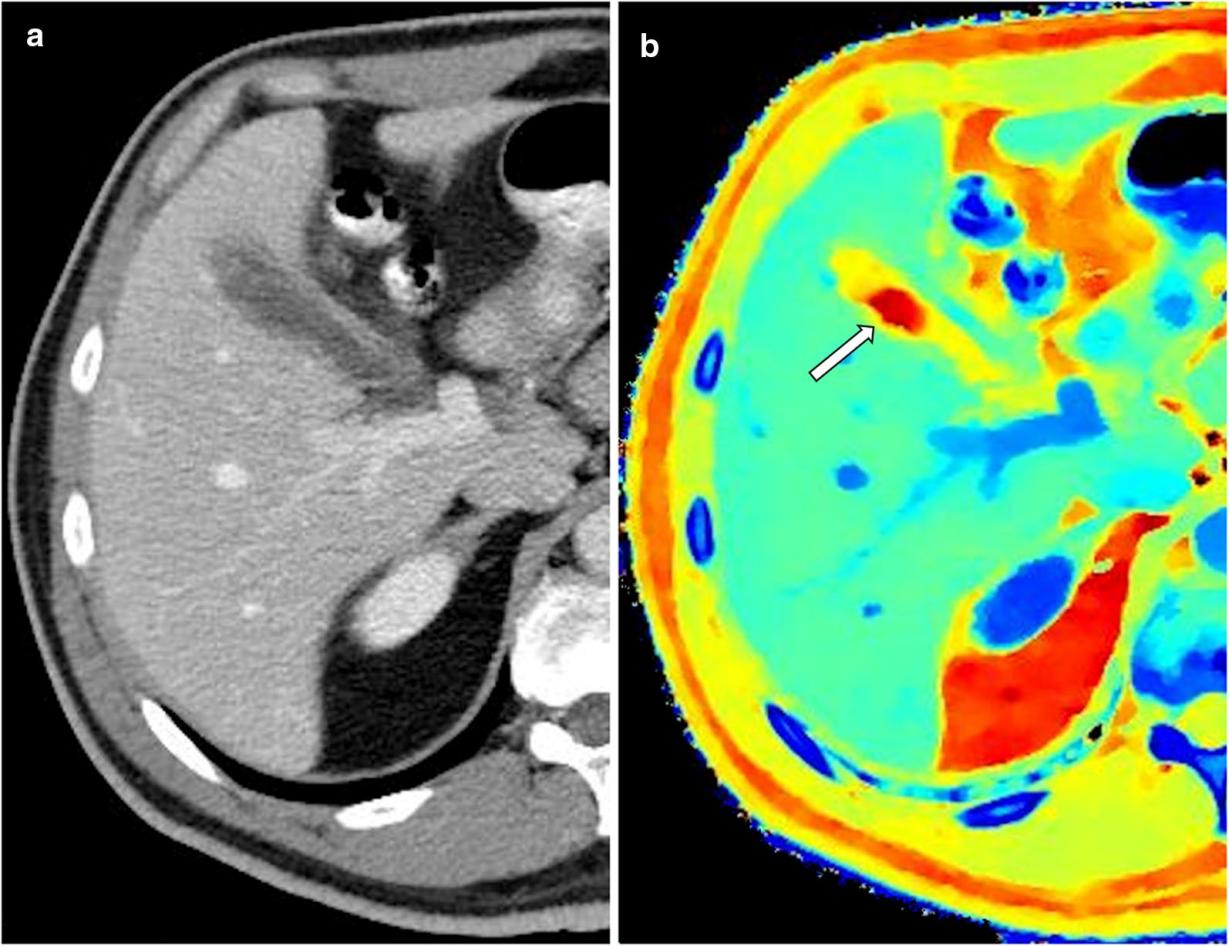
CT of a 64-year-old man showing mild heterogeneity of the gallbladder contents on conventional images (**a**), with a clear gallbladder stone visible on the effective atomic number map (**b**) as a focal area with lower atomic number than surrounding bile (arrow)

Gallstones are often not identified on conventional CT, with more than half being radiolucent [60]. Thus, patients who undergo CT scanning for various reasons often require further workup on ultrasound or magnetic resonance cholangiopancreatography (MRCP), or even diagnostic endoscopic retrograde cholangiopancreatography (ERCP), depending on the clinical scenario. If CT could visualize cholelithiasis and choledocholithiasis reliably, there will be significant savings in cost and time-to-diagnosis. Spectral material decomposition images detect non-calcified stones with high accuracy, because stones have a slightly lower effective atomic number (Z-effective) than bile (Fig. 7), and have a lower attenuation than bile on low keV images, optimally seen on 40 keV, and higher attenuation than bile on high keV images, optimally 140 keV [60, 61].

Acute cholecystitis is usually first evaluated on ultrasound, although large variability in sensitivity is seen with ultrasound [62], because of its wide availability and better visualization of gallstones, in conjunction with the ability to evaluate for a sonographic Murphy sign. However, studies have shown that CT in fact has better sensitivity [62, 63]. Spectral CT using low keV images and iodine maps may even improve the sensitivity for diagnosis, and can better evaluate suspected gangrenous cholecystitis because of better visualization of gallbladder wall enhancement abnormalities [60].

### Kidneys

**Fig. 8:**
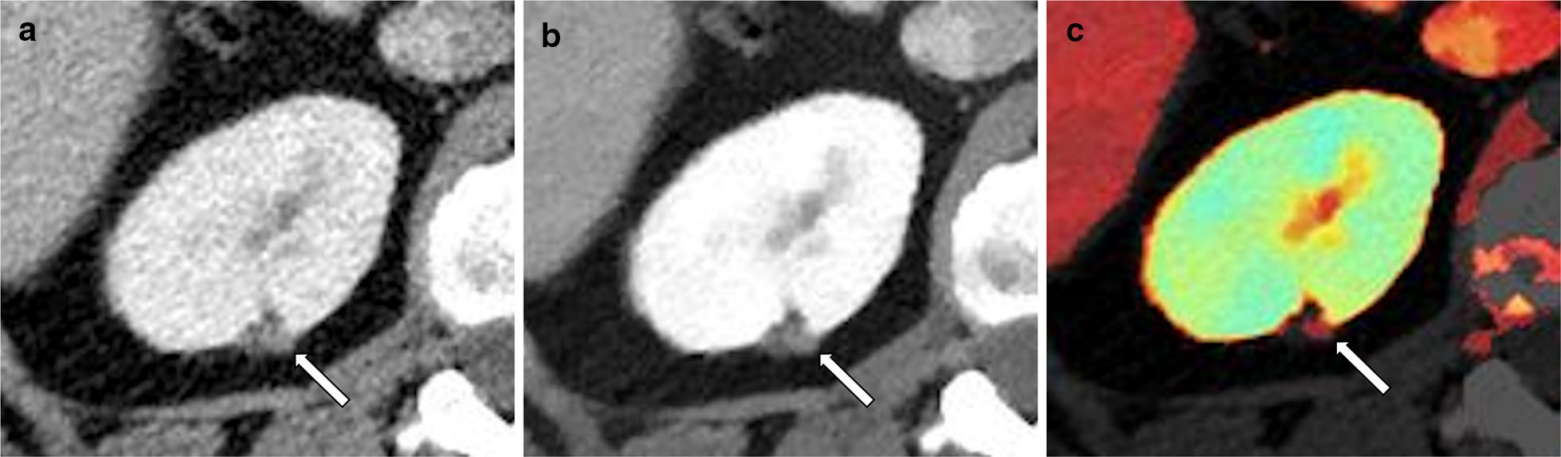
A 54-year-old woman underwent a routine abdominal CT before hernioplasty. Conventional images showed an incidental finding in the right kidney (arrows) with heterogeneous attenuation on the portovenous phase (**a**). The lesion’s heterogeneity and suspected enhancing component are accentuated using a 50 keV mono-energetic image (**b**). Iodine maps prove that the lesion’s high attenuation is true enhancement (**c**), showing iodine content of 4.8 mg/mL and a normalized iodine content of 1.1 mg/mL, suspicious for RCC

Incidental renal lesions are common, seen in approximately 10% of CT scans. While most are simple cysts, it is often difficult to distinguish between benign complicated cysts, possibly malignant complicated cysts and solid lesions on a single phase contrast enhanced study. This diagnostic dilemma leads to many follow up studies both on CT and MR. Spectral CT may enable accurate evaluation of some of these lesions, thus decreasing diagnostic costs and time. This has been one of the earliest applications of spectral CT, due to the high incidence of indeterminate renal incidentalomas [26, 34, 64–68]. Spectral CT can differentiate non-enhancing from enhancing lesions on single-phase scans (Fig. 8) using VNC reconstructions and also iodine maps and spectral attenuation curves [69], with the diagnosis of avidly enhancing lesions such as clear-cell renal cell carcinoma (RCC) being easier than the less avidly enhancing papillary RCC. The threshold for determining enhancement of a lesion has been studied, and differences have been found between different spectral techniques and different scan timings (arterial vs. nephrographic) [34, 64]. For rapid kV switching, the reported thresholds are 1.22–2.0 ng/mL, while for dual source scanners the reported thresholds are 0.5–1.3 ng/mL. Dual layer scanners thresholds have not been published yet. Normalizing the thresholds to the aorta's measured iodine concentration reduces intra-manufacturer variability, with a threshold of 0.3 across platforms [34]. Effective atomic number can also be used to diagnose enhancing masses, with an optimal threshold of 8.36 yielding a diagnostic accuracy of 86.6%[66]. Measuring iodine concentration is at least as accurate as measuring enhancement values on enhanced images compared with unenhanced images [65, 67, 68], but the effect of pseudo-enhancement on accurate measurements must still be considered as it is with conventional CT [70, 71]. This is most pronounced in small lesions, with a diameter under 8 mm, which is why there is likely still a size threshold for reliably evaluating renal lesions, even with spectral imaging. In these cases VNC has similar limitations when there is insufficient iodine removal close to the collecting system or when there is avid enhancement of the renal parenchyma [69].

Iodine concentration is also showing promise in differentiating between histological sub-types of RCC, and has been shown to accurately differentiate between clear-cell RCC and papillary RCC, using a threshold of 0.9 mg/mL with an overall accuracy of 95.3%, while also showing promise in determining tumor grade [72].

Low keV mono-energetic images can also be used to decrease the amount of contrast used in CT urography, with adequate enhancement achieved using 50% of contrast material dose both for urographic phase evaluation and for blood vessel evaluation prior to surgery [73].

**Fig. 9:**
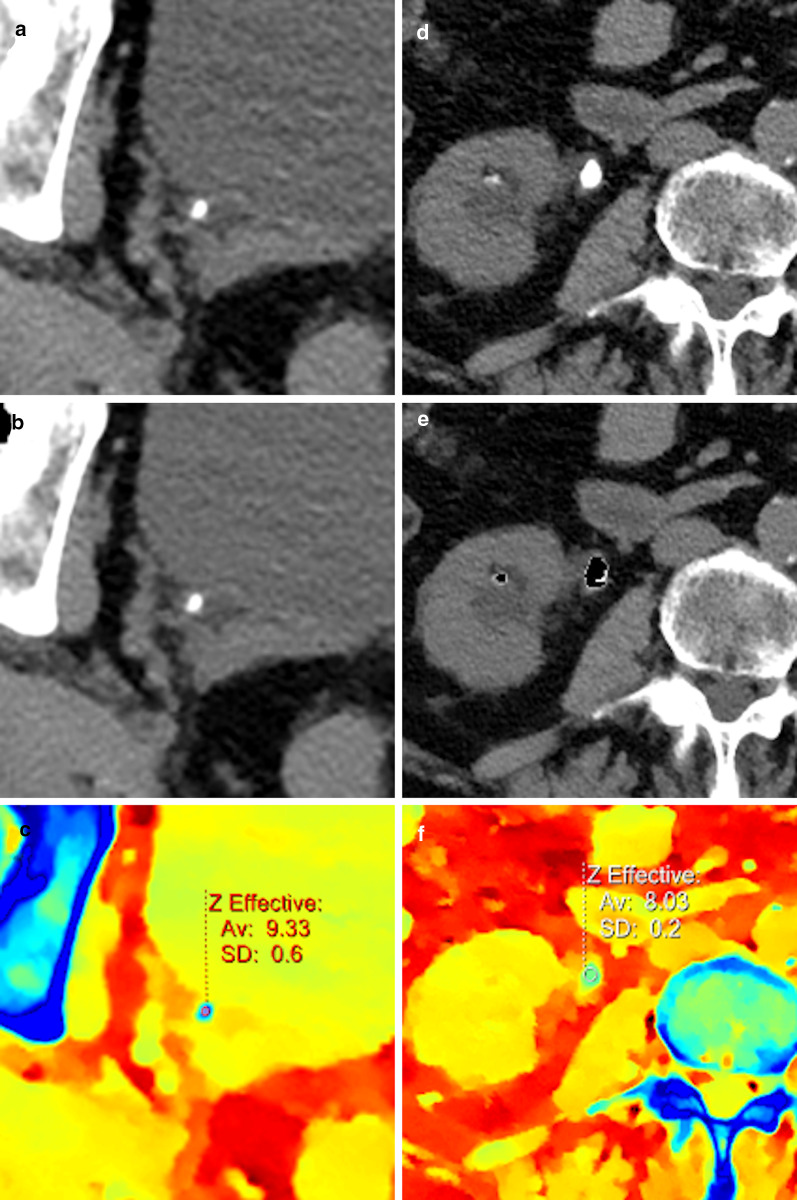
Side-by-side images showing uric acid evaluation in two different patients evaluated for nephrolithiasis. **a**–**c** showing a calcium-containing stone in the distal ureter, (**d**–**f**) showing uric acid stones in the lower pole calix and proximal ureter, proven as uric acid stones by laboratory evaluation following lithotripsy and stenting. **a**, **d** conventional anatomic images, (**b**, **e**) "uric acid removed" overlay images, on which uric acid is removed from the image leaving a black pixels, and (**c**, **f**) are atomic number images showing the different atomic numbers of the different types of stones

Evaluation of nephrolithiasis has also been markedly improved with spectral imaging. Uric acid stones, which are often treated medically, can be reliably distinguished from other types of stones (Fig. 9), which may otherwise necessitate an invasive approach, with an accuracy ranging between 90 to 100% [58, 60, 74, 75]. More advanced material decomposition using dual source systems with tin filtration, or advanced applications of spectral detectors, can also differentiate between most other types of stones [60, 76], although this has a lesser clinical impact.

### Adrenals

Adrenal incidentalomas, seen in 1–4.2% of the scanned population, are considered a major cause of further imaging required for incidental lesions, and have widely acceptable imaging guidelines on conventional CT [77]. Adenomas can be diagnosed on conventional CT using TNC images, with a threshold of 10 HU for differentiating between adenomas and other lesions, and with dedicated adrenal scans using washout calculations [77]. However, single phase enhanced routine abdominal scans cannot make this differentiation, leading to further imaging. Spectral imaging has the potential to obviate further imaging, both by enabling diagnosis on single phase enhanced CT scans, and by improving the diagnosis of lesions considered to be indeterminate, using current CT and MRI criteria.

Spectral VNC images can be used instead of TNC, although the minimal difference in HU measurements between VNC and TNC [11, 78, 79] leads to altered sensitivity and specificity when using the same threshold, and the optimal VNC threshold for diagnosing an adenoma has not been determined yet. However, it can be noted that no false positive cases were found when using the same threshold [80]. This implies this threshold can be used to safely diagnose adenomas, with the risk of calling more lesions indeterminate compared to TNC (Fig. 2). Studies have also shown differences in attenuation of adrenal lesions on VNC compared to TNC images, reported more when scans were performed early in the portal-venous phase compared to later in the portal-venous phase [80]. Therefore, reaching a widely accepted threshold for diagnosing adenomas on VNC using routine abdominal scans which are likely to be performed with slightly different scan timings in different institutions may prove to be difficult.

Studies have also demonstrated the superiority of spectral derived data other than VNC, using attenuation curves at different keV, material decomposition using fat–water and fat-iodine pairs, z-effective and other parameters [26, 64, 81]. These studies need further corroboration using larger cohorts and multiple spectral technologies, before the results can be used in clinical practice.

### Gastrointestinal tract

**Fig. 10:**
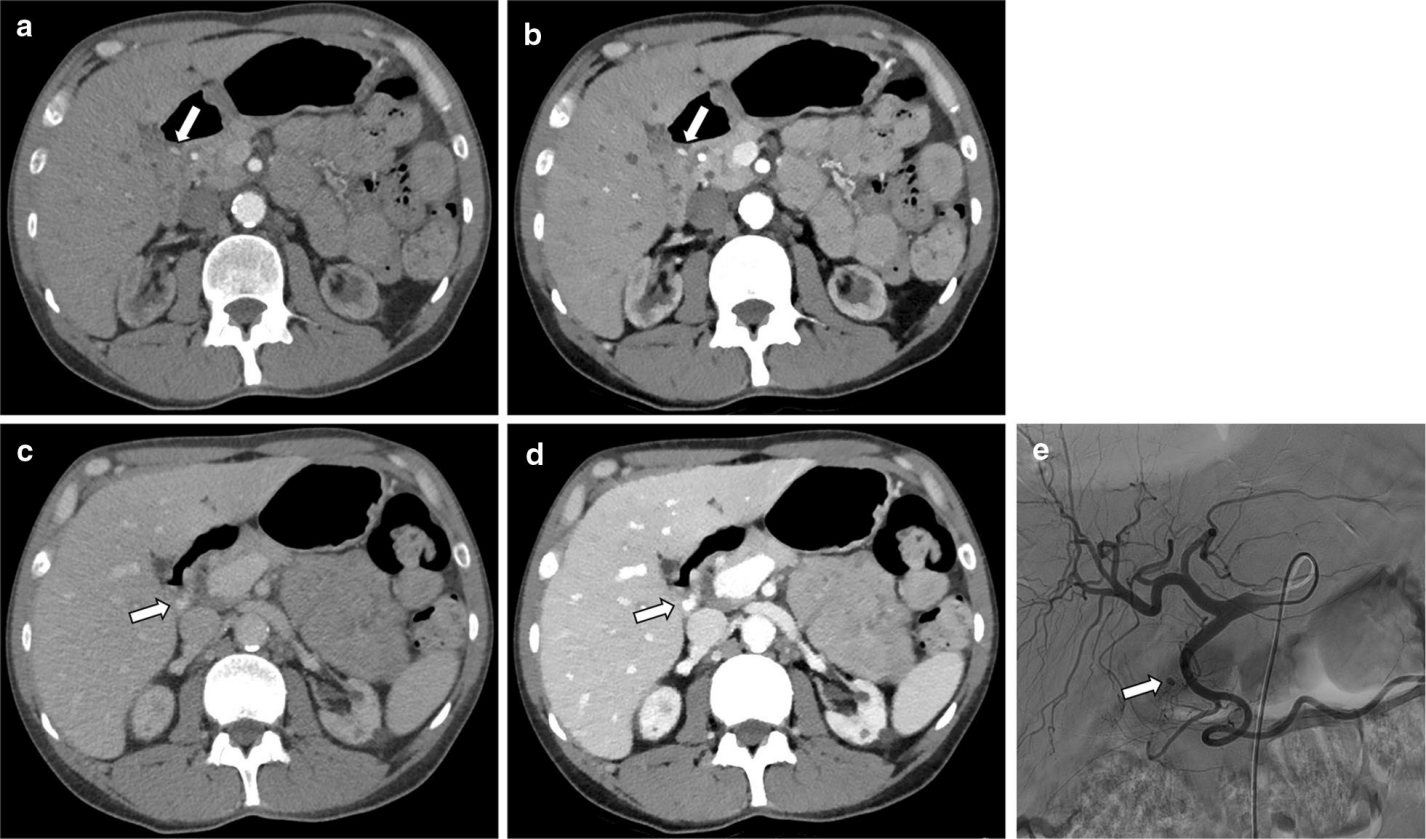
A 40-year-old man with upper gastrointestinal bleeding who underwent multiphasic CT for localization. Conventional images show contrast extravasation in the duodenal bulb (arrows) on the arterial (**a**) and venous (**c**) phases. The extravasation is more conspicuous using 50 keV mono-energetic images on both the arterial (**b**) and venous (**d**) phases. The focus of active bleeding is proven on digital subtraction angiography (**e**)

The obvious application in evaluating bowel disease is the evaluation of active hemorrhage. Low keV images accentuate active extravasation and may facilitate detection of intraluminal hemorrhage (Fig. 10) [60, 82]. The use of VNC images in lieu of TNC images also has the potential to reduce radiation in active gastrointestinal hemorrhage protocols [40] and allow for confident detection of hemorrhage on non-dedicated portal-venous phase only scans.

Acute ischemia may also be better evaluated on spectral CT, in both acute mesenteric occlusion [83, 84] and in ischemia secondary to bowel obstruction [85, 86]. Although one study has only been able to show increased diagnostic confidence, without improved diagnostic performance [86], the other studies have shown improved performance for detection of abnormally enhancing bowel segments using iodine maps and low keV images.

**Fig. 11:**
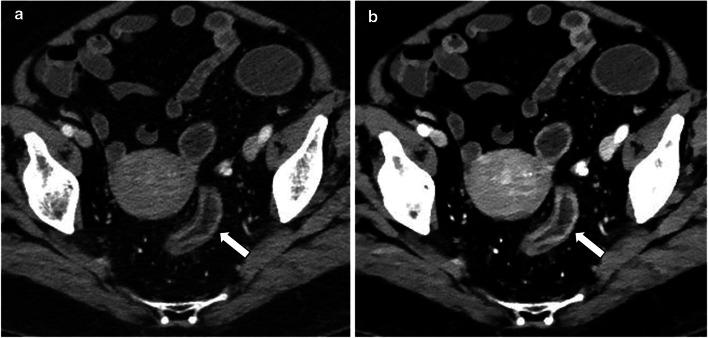
CT enterography of a 74-year-old woman with known Crohn's disease of the colon. Conventional images show mild mucosal hyperenhancement in the sigmoid colon (**a**) which is more conspicuous using 50 keV mono-energetic images (**b**)

Active bowel inflammation severity in Crohn's disease has been shown to be related to iodine concentration [87, 88] and spectral HU curves [88]. Qualitative assessment of active inflammation may also be improved because of the heightened hyper-enhancement, seen on low keV images (Fig. 11). The most useful low keV setting for diagnosis of active inflammation was found to be 40 keV (the lowest achievable in the study) [89], surprising considering the fact that this keV also has the greatest amount of noise and is not routinely used in clinical practice in most centers [86]. Acute appendicitis, which is another type of inflammation, can also become more visible using iodine maps and low keV images [60] and these images also improve diagnostic performance in cases of gangrenous appendicitis [90].

CT colonography uses low-dose CT to evaluate the colon for polyps, and electronic fecal cleansing is often used to correct for inadequate bowel preparation, by using fecal tagging with iodine or barium. Electronic cleansing often causes artifacts which decrease image quality. Spectral data allows for better electronic cleansing and fecal tagging, with improved image quality [91, 92]. Deep learning is now being used to improve cleansing algorithms, and has been shown to work better when using spectral data [93].

Bowel tumor evaluation on spectral CT is also being investigated. The obvious advantages lie in the detection of primary or metastatic hyper-enhancing tumors, such as neuroendocrine tumors and melanoma metastases using low KeV mono-energetic images and iodine maps [94]. Spectral data is also showing promise in determining histological origin and grade, for instance determining the histological origin of ampullary tumors [95], differentiating between bowel adenocarcinoma and lymphoma [96], differentiating squamous cell vs. adenocarcinoma of the gastroesophageal junction [97], differentiating high vs low-risk gastrointestinal stromal tumors (GIST) [98], diagnosing colorectal carcinomas with microsatellite instability [50] or high grade characteristics [99]. Differentiating metastatic lymph nodes from benign lymph nodes may also be possible using z-effective and iodine concentrations, with accuracy similar to MRI on one study, and this has been studied mostly for rectal carcinoma but also gastric cancers [100–102]. Response to treatment may also be better evaluated with spectral data, as shown in rectal tumors [103] and in GIST [64].

### Genital system

Although not extensively studied, the same basic principles as in other organs apply to the genital system. A major possible application is differentiation between benign and malignant tumors. Iodine maps and low KeV images improve the identification of enhancing septations, which raise the suspicion for malignancy [104]. A recent study found an iodine content threshold of 0.9 mg/mL to have an 81% sensitivity and a 73% specificity in distinguishing benign from malignant tumors. It also found that a Z-effective threshold of 8.16 had an 85% sensitivity and a 73% specificity [105]. For assessment of depth of myometrial invasion of endometrial carcinoma, low KeV images perform comparably to trans-vaginal ultrasound, when compared with pathology, with a 91% sensitivity, 100% specificity and 94% accuracy, with conventional-equivalent images showing only a 57% sensitivity, 86% specificity and 71% accuracy [106]. In cervical malignancies, iodine maps are showing promise in evaluating response to chemo-radiation therapy and in predicting outcome [107]. Iodine content has also shown promise in differentiating cervical carcinoma involvement of lymph nodes from normal or reactive lymph nodes [108], with metastatic lymph nodes showing significantly lower iodine content, although no definitive thresholds exist.

## Conclusions and future developments

Currently, the most well established application of spectral imaging is the use of mono-energetic images of high and low KeV for better visualization of enhancing structures, decreasing contrast media volume and decreasing metal artifacts. VNC and z-effective evaluation of specific materials are also relatively robust and can be used clinically in certain settings, but need further improvement before widely adopted, in our opinion. Using spectral data for lesion characterization is very promising, however, we believe there is not enough knowledge to create a clinically robust threshold for iodine content. There is also variability derived from the different hardware and algorithms used by the different vendors, possibly precluding our ability to reach cross-platform thresholds. This application will likely become well established for clinical use only when such universal thresholds are determined.

The future of spectral CT is right beyond the bend, with photon-counting systems already being developed and site-tested. These scanners are expected to be available clinically in the next few years, and are expected to improve material specificity, energy separation and likely also inter-vendor variability.

## Data Availability

Not applicable (this is a review, not original research).

## Abbreviations

CNR: Contrast-to-noise ratio
CT: Computed tomography
ERCP: Endoscopic retrograde cholangiopancreatography
GIST: Gastrointestinal stromal tumors
HCC: Hepatocellular carcinoma
HU: Hounsfield units
keV: Kilo-electron Volt
kV: Kilo-Volt
kVp: Peak tube voltage
MRCP: Magnetic resonance cholangiopancreatography
MRI: Magnetic Resonance Imaging
NET: Neuroendocrine tumor
RCC: Renal cell carcinoma
SNR: Signal-to-noise ratio
TNC: True non-contrast
VNC: Virtual non-contrast

## References

[1] Krauss B (2018). Dual-energy computed tomography: technology and challenges. Radiol Clin North Am.

[2] Jacobsen M, Schellingerhout D, Wood C (2017). Intermanufacturer comparison of dual-energy CT iodine quantification and monochromatic attenuation: a phantom study. Radiology.

[3] Megibow AJ, Kambadakone A, Ananthakrishnan L (2018). Dual-energy computed tomography: image acquisition, processing, and workflow. Radiol Clin North Am.

[4] van Ommen F, de Jong HWAM, Dankbaar JW (2019). Dose of CT protocols acquired in clinical routine using a dual-layer detector CT scanner: a preliminary report. Eur J Radiol.

[5] Siegel MJ, Mhlanga JC, Salter A, Ramirez-Giraldo JC (2021). Comparison of radiation dose and image quality between contrast-enhanced single- and dual-energy abdominopelvic computed tomography in children as a function of patient size. Pediatr Radiol.

[6] Forghani R, De Man B, Gupta R (2017). Dual-energy computed tomography: physical principles, approaches to scanning, usage, and implementation: part 1. Neuroimaging Clin N Am.

[7] Jamali S, Michoux N, Coche E, Dragean CA (2019). Virtual unenhanced phase with spectral dual-energy CT: is it an alternative to conventional true unenhanced phase for abdominal tissues?. Diagn Interv Imaging.

[8] Parakh A, Macri F, Sahani D (2018). Dual-energy computed tomography: dose reduction, series reduction, and contrast load reduction in dual-energy computed tomography. Radiol Clin North Am.

[9] Ananthakrishnan L, Rajiah P, Ahn R (2017). Spectral detector CT-derived virtual non-contrast images: comparison of attenuation values with unenhanced CT. Abdom Radiol (NY).

[10] Durieux P, Gevenois PA, Van MA (2018). Abdominal attenuation values on virtual and true unenhanced images obtained with third-generation dual-source dual-energy CT. AJR Am J Roentgenol.

[11] Slebocki K, Kraus B, Chang D-H (2017). Incidental Findings in Abdominal Dual-Energy Computed Tomography: Correlation Between True Noncontrast and Virtual Noncontrast Images Considering Renal and Liver Cysts and Adrenal Masses. J Comput Assist Tomogr.

[12] Grosse Hokamp N, Gilkeson R, Jordan MK (2019). Virtual monoenergetic images from spectral detector CT as a surrogate for conventional CT images: Unaltered attenuation characteristics with reduced image noise. Eur J Radiol.

[13] Atwi NE, Smith DL, Flores CD (2019). Dual-energy CT in the obese: a preliminary retrospective review to evaluate quality and feasibility of the single-source dual-detector implementation. Abdom Radiol (NY).

[14] Matsumoto K, Jinzaki M, Tanami Y (2011). Virtual monochromatic spectral imaging with fast kilovoltage switching: improved image quality as compared with that obtained with conventional 120-kVp CT. Radiology.

[15] Wei L, Li S, Gao Q (2016). Use of low tube voltage and low contrast agent concentration yields good image quality for aortic CT angiography. Clin Radiol.

[16] Higashigaito K, Schmid T, Puippe G (2016). CT Angiography of the Aorta: Prospective Evaluation of Individualized Low-Volume Contrast Media Protocols. Radiology.

[17] Ippolito D, Talei Franzesi C, Fior D (2015). Low kV settings CT angiography (CTA) with low dose contrast medium volume protocol in the assessment of thoracic and abdominal aorta disease: a feasibility study. Br J Radiol.

[18] Chung YE, You JS, Lee H-J (2015). Possible contrast media reduction with low keV monoenergetic images in the detection of focal liver lesions: a dual-energy CT animal study. PLoS One.

[19] Shuman WP, O’Malley RB, Busey JM (2017). Prospective comparison of dual-energy CT aortography using 70% reduced iodine dose versus single-energy CT aortography using standard iodine dose in the same patient. Abdom Radiol (NY).

[20] Hickethier T, Kroeger JR, Lennartz S (2019). Venous-phase chest CT with reduced contrast medium dose: Utilization of spectral low keV monoenergetic images improves image quality. Eur J Radiol.

[21] Noda Y, Goshima S, Nakashima Y (2019). Iodine dose optimization in portal venous phase virtual monochromatic images of the abdomen: Prospective study on rapid kVp switching dual energy CT. Eur J Radiol.

[22] Tsang DS, Merchant TE, Merchant SE (2017). Quantifying potential reduction in contrast dose with monoenergetic images synthesized from dual-layer detector spectral CT. Br J Radiol.

[23] Clark ZE, Bolus DN, Little MD, Morgan DE (2015). Abdominal rapid-kVp-switching dual-energy MDCT with reduced IV contrast compared to conventional MDCT with standard weight-based IV contrast: an intra-patient comparison. Abdom Imaging.

[24] Parakh A, Negreros-Osuna AA, Patino M (2019). Low-keV and Low-kVp CT for Positive Oral Contrast Media in Patients with Cancer: A Randomized Clinical Trial. Radiology.

[25] Patino M, Murcia DJ, Iamurri AP (2017). Impact of low-energy CT imaging on selection of positive oral contrast media concentration. Abdom Radiol (NY).

[26] Fulton N, Rajiah P (2018). Abdominal applications of a novel detector-based spectral CT. Curr Probl Diagn Radiol.

[27] Wellenberg RHH, Boomsma MF, van Osch JAC (2017). Quantifying metal artefact reduction using virtual monochromatic dual-layer detector spectral CT imaging in unilateral and bilateral total hip prostheses. Eur J Radiol.

[28] Hakvoort ET, Wellenberg RHH, Streekstra GJ (2019). Quantifying near metal visibility using dual energy computed tomography and iterative metal artifact reduction in a fracture phantom. Phys Med.

[29] Lu X, Lu Z, Yin J, et al (2019) Effects of radiation dose levels and spectral iterative reconstruction levels on the accuracy of iodine quantification and virtual monochromatic CT numbers in dual-layer spectral detector CT: an iodine phantom study. Quant Imaging Med Surg 9:188–200. 10.21037/qims.2018.11.12 10.21037/qims.2018.11.12 PMC6414759 30976543

[30] Kim H, Park CM, Kang CK (2018). Effect of CT acquisition parameters on iodine density measurement at dual-layer spectral CT. AJR Am J Roentgenol.

[31] Wortman JR, Sodickson AD (2018). Pearls, pitfalls, and problems in dual-energy computed tomography imaging of the body. Radiol Clin North Am.

[32] Jacobsen MC, Cressman ENK, Tamm EP (2019). Dual-energy CT: lower limits of iodine detection and quantification. Radiology.

[33] Grosse Hokamp N, Abdullayev N, Persigehl T (2019). Precision and reliability of liver iodine quantification from spectral detector CT: evidence from phantom and patient data. Eur Radiol.

[34] Patel BN, Vernuccio F, Meyer M (2019). Dual-energy CT material density iodine quantification for distinguishing vascular from nonvascular renal lesions: normalization reduces intermanufacturer threshold variability. AJR Am J Roentgenol.

[35] Boning G, Feldhaus F, Adelt S (2019). Clinical routine use of virtual monochromatic datasets based on spectral CT in patients with hypervascularized abdominal tumors - evaluation of effectiveness and efficiency. Acta Radiol.

[36] Park JH, Kim SH, Park HS (2011). Added value of 80 kVp images to averaged 120 kVp images in the detection of hepatocellular carcinomas in liver transplantation candidates using dual-source dual-energy MDCT: results of JAFROC analysis. Eur J Radiol.

[37] Lv P, Lin XZ, Chen K, Gao J (2012). Spectral CT in patients with small HCC: investigation of image quality and diagnostic accuracy. Eur Radiol.

[38] Anzidei M, Di Martino M, Sacconi B (2015). Evaluation of image quality, radiation dose and diagnostic performance of dual-energy CT datasets in patients with hepatocellular carcinoma. Clin Radiol.

[39] Grosse Hokamp N, Obmann VC, Kessner R (2018). Improved visualization of hypodense liver lesions in virtual monoenergetic images from spectral detector CT: Proof of concept in a 3D-printed phantom and evaluation in 74 patients. Eur J Radiol.

[40] Sun H, Hou X-Y, Xue H-D (2015). Dual-source dual-energy CT angiography with virtual non-enhanced images and iodine map for active gastrointestinal bleeding: image quality, radiation dose and diagnostic performance. Eur J Radiol.

[41] Ascenti G, Sofia C, Mazziotti S (2016). Dual-energy CT with iodine quantification in distinguishing between bland and neoplastic portal vein thrombosis in patients with hepatocellular carcinoma. Clin Radiol.

[42] Su L, Hu L, Liang P (2019). Clinical efficacy of spectral computed tomography for evaluating liver function in patients with Budd–Chiari syndrome. Acad Radiol.

[43] Wang N, Ju Y, Wu J (2019). Differentiation of liver abscess from liver metastasis using dual-energy spectral CT quantitative parameters. Eur J Radiol.

[44] Pfeiffer D, Parakh A, Patino M (2018). Iodine material density images in dual-energy CT: quantification of contrast uptake and washout in HCC. Abdom Radiol (NY).

[45] Gao L, Lv Y, Jin Y (2019). Differential diagnosis of hepatic cancerous nodules and cirrhosis nodules by spectral CT imaging: a feasibility study. Acta Radiol.

[46] Bottari A, Silipigni S, Carerj ML (2019). Dual-source dual-energy CT in the evaluation of hepatic fractional extracellular space in cirrhosis. Radiol Med.

[47] Hyodo T, Yada N, Hori M (2017). Multimaterial decomposition algorithm for the quantification of liver fat content by using fast-kilovolt-peak switching dual-energy CT: clinical evaluation. Radiology.

[48] Hyodo T, Hori M, Lamb P (2017). Multimaterial decomposition algorithm for the quantification of liver fat content by using Fast–Kilovolt–Peak switching dual-energy CT: experimental validation. Radiology.

[49] Zheng X, Ren Y, Phillips WT (2013). Assessment of hepatic fatty infiltration using spectral computed tomography imaging: a pilot study. J Comput Assist Tomogr.

[50] Wu J, Lv Y, Wang N (2019). The value of single-source dual-energy CT imaging for discriminating microsatellite instability from microsatellite stability human colorectal cancer. Eur Radiol.

[51] Kramer H, Pickhardt PJ, Kliewer MA (2017). Accuracy of liver fat quantification with advanced CT, MRI, and ultrasound techniques: prospective comparison with MR spectroscopy. AJR Am J Roentgenol.

[52] Zhang YN, Fowler KJ, Hamilton G (2018). Liver fat imaging-a clinical overview of ultrasound, CT, and MR imaging. Br J Radiol.

[53] El Kayal N, Lennartz S, Ekdawi S (2019). Value of spectral detector computed tomography for assessment of pancreatic lesions. Eur J Radiol.

[54] Nagayama Y, Tanoue S, Inoue T (2019). Dual-layer spectral CT improves image quality of multiphasic pancreas CT in patients with pancreatic ductal adenocarcinoma. Eur Radiol.

[55] Beer L, Toepker M, Ba-Ssalamah A (2019). Objective and subjective comparison of virtual monoenergetic vs. polychromatic images in patients with pancreatic ductal adenocarcinoma. Eur Radiol.

[56] Noda Y, Goshima S, Kaga T (2019). Virtual monochromatic image at lower energy level for assessing pancreatic ductal adenocarcinoma in fast kV-switching dual-energy CT. Clin Radiol.

[57] Martin SS, Trapp F, Wichmann JL (2019). Dual-energy CT in early acute pancreatitis: improved detection using iodine quantification. Eur Radiol.

[58] Mohammed MF, Elbanna KY, Mohammed AME (2018). Practical applications of dual-energy computed tomography in the acute abdomen. Radiol Clin North Am.

[59] Yin Q, Zou X, Zai X (2015). Pancreatic ductal adenocarcinoma and chronic mass-forming pancreatitis: Differentiation with dual-energy MDCT in spectral imaging mode. Eur J Radiol.

[60] Murray N, Darras KE, Walstra FE (2019). Dual-Energy CT in Evaluation of the Acute Abdomen. Radiographics.

[61] Li H, He D, Lao Q (2015). Clinical value of spectral CT in diagnosis of negative gallstones and common bile duct stones. Abdom Imaging.

[62] Kiewiet JJS, Leeuwenburgh MMN, Bipat S (2012). A systematic review and meta-analysis of diagnostic performance of imaging in acute cholecystitis. Radiology.

[63] Wertz JR, Lopez JM, Olson D, Thompson WM (2018). Comparing the diagnostic accuracy of ultrasound and CT in evaluating acute cholecystitis. AJR Am J Roentgenol.

[64] Morgan DE (2018). The role of dual-energy computed tomography in assessment of abdominal oncology and beyond. Radiol Clin North Am.

[65] Ascenti G, Mileto A, Krauss B (2013). Distinguishing enhancing from nonenhancing renal masses with dual-source dual-energy CT: iodine quantification versus standard enhancement measurements. Eur Radiol.

[66] Mileto A, Allen BC, Pietryga JA (2017). Characterization of incidental renal mass with dual-energy CT: diagnostic accuracy of effective atomic number maps for discriminating nonenhancing cysts from enhancing masses. AJR Am J Roentgenol.

[67] Mileto A, Marin D, Ramirez-Giraldo JC (2014). Accuracy of contrast-enhanced dual-energy MDCT for the assessment of iodine uptake in renal lesions. AJR Am J Roentgenol.

[68] Bellini D, Panvini N, Laghi A (2019). Systematic review and meta-analysis investigating the diagnostic yield of dual-energy CT for renal mass assessment. AJR Am J Roentgenol.

[69] Mileto A, Sofue K, Marin D (2016). Imaging the renal lesion with dual-energy multidetector CT and multi-energy applications in clinical practice: what can it truly do for you?. Eur Radiol.

[70] Soesbe TC, Ananthakrishnan L, Lewis MA (2018). Pseudoenhancement effects on iodine quantification from dual-energy spectral CT systems: a multi-vendor phantom study regarding renal lesion characterization. Eur J Radiol.

[71] Patel BN, Farjat A, Schabel C (2018). Energy-specific optimization of attenuation thresholds for low-energy virtual monoenergetic images in renal lesion evaluation. AJR Am J Roentgenol.

[72] Mileto A, Marin D, Alfaro-Cordoba M (2014). Iodine quantification to distinguish clear cell from papillary renal cell carcinoma at dual-energy multidetector CT: a multireader diagnostic performance study. Radiology.

[73] Shuman WP, Mileto A, Busey JM (2019). Dual-energy CT urography With 50% reduced iodine dose versus single-energy CT urography with standard iodine dose. AJR Am J Roentgenol.

[74] Ananthakrishnan L, Duan X, Xi Y (2018). Dual-layer spectral detector CT: non-inferiority assessment compared to dual-source dual-energy CT in discriminating uric acid from non-uric acid renal stones ex vivo. Abdom Radiol (NY).

[75] Lombardo F, Bonatti M, Zamboni GA (2017). Uric acid versus non-uric acid renal stones: in vivo differentiation with spectral CT. Clin Radiol.

[76] Grosse Hokamp N, Salem J, Hesse A (2018). Low-dose characterization of kidney stones using spectral detector computed tomography: an ex vivo study. Invest Radiol.

[77] Adam SZ, Nikolaidis P, Horowitz JM (2016). Chemical shift MR imaging of the adrenal gland: principles, pitfalls, and applications. Radiographics.

[78] Helck A, Hummel N, Meinel FG (2014). Can single-phase dual-energy CT reliably identify adrenal adenomas?. Eur Radiol.

[79] Botsikas D, Triponez F, Boudabbous S (2014). Incidental adrenal lesions detected on enhanced abdominal dual-energy CT: can the diagnostic workup be shortened by the implementation of virtual unenhanced images?. Eur J Radiol.

[80] Connolly MJ, McInnes MDF, El-Khodary M (2017). Diagnostic accuracy of virtual non-contrast enhanced dual-energy CT for diagnosis of adrenal adenoma: a systematic review and meta-analysis. Eur Radiol.

[81] Ju Y, Liu A, Dong Y (2015). The value of nonenhanced single-source dual-energy CT for differentiating metastases from adenoma in adrenal glands. Acad Radiol.

[82] Liu W-D, Wu X-W, Hu J-M (2015). Monochromatic energy computed tomography image for active intestinal hemorrhage: a model investigation. World J Gastroenterol.

[83] Potretzke TA, Brace CL, Lubner MG (2015). Early small-bowel ischemia: dual-energy CT improves conspicuity compared with conventional CT in a swine model. Radiology.

[84] Lourenco PDM, Rawski R, Mohammed MF (2018). Dual-energy CT iodine mapping and 40-keV monoenergetic applications in the diagnosis of acute bowel ischemia. AJR Am J Roentgenol.

[85] Oda S, Nakaura T, Utsunomiya D (2017). Clinical potential of retrospective on-demand spectral analysis using dual-layer spectral detector-computed tomography in ischemia complicating small-bowel obstruction. Emerg Radiol.

[86] Darras KE, McLaughlin PD, Kang H (2016). Virtual monoenergetic reconstruction of contrast-enhanced dual energy CT at 70keV maximizes mural enhancement in acute small bowel obstruction. Eur J Radiol.

[87] Kim YS, Kim SH, Ryu HS, Han JK (2018). Iodine quantification on spectral detector-based dual-energy CT enterography: correlation with Crohn’s disease activity index and external validation. Korean J Radiol.

[88] Peng JC, Feng Q, Zhu J (2016). Usefulness of spectral computed tomography for evaluation of intestinal activity and severity in ileocolonic Crohn’s disease. Therap Adv Gastroenterol.

[89] Lee SM, Kim SH, Ahn SJ (2018). Virtual monoenergetic dual-layer, dual-energy CT enterography: optimization of keV settings and its added value for Crohn’s disease. Eur Radiol.

[90] Elbanna KY, Mohammed MF, Chahal T (2018). Dual-energy CT in differentiating nonperforated gangrenous appendicitis from uncomplicated appendicitis. AJR Am J Roentgenol.

[91] Eliahou R, Azraq Y, Carmi R (2010). Dual-energy based spectral electronic cleansing in non-cathartic computed tomography colonography: an emerging novel technique. Semin Ultrasound CT MR.

[92] Taguchi N, Oda S, Imuta M (2018). Dual-energy computed tomography colonography using dual-layer spectral detector computed tomography: Utility of virtual monochromatic imaging for electronic cleansing. Eur J Radiol.

[93] Tachibana R, Näppi JJ, Ota J, et al (2018) Deep Learning Electronic Cleansing for Single- and Dual-Energy CT Colonography. Radiographics 38:2034–2050. 10.1148/rg.2018170173 10.1148/rg.2018170173 PMC6276077 30422761

[94] Yeh BM, Obmann MM, Westphalen AC (2018). Dual energy computed tomography scans of the bowel: benefits, pitfalls, and future directions. Radiol Clin North Am.

[95] Wei W, Yu Y, Lv W (2014). Predictive value of dual-energy spectral computed tomographic imaging on the histological origin of carcinomas in the ampullary region. Abdom Imaging.

[96] Yang C-B, Yu N, Jian Y-J (2019). Spectral CT imaging in the differential diagnosis of small bowel adenocarcinoma from primary small intestinal lymphoma. Acad Radiol.

[97] Zhou Y, Hou P, Zha K (2019). Spectral computed tomography for the quantitative assessment of patients with carcinoma of the gastroesophageal junction: initial differentiation between a diagnosis of squamous cell carcinoma and adenocarcinoma. J Comput Assist Tomogr.

[98] Zhang X, Bai L, Wang D (2019). Gastrointestinal stromal tumor risk classification: spectral CT quantitative parameters. Abdom Radiol (NY).

[99] Gong H-X, Zhang K-B, Wu L-M (2016). Dual energy spectral CT imaging for colorectal cancer grading: a preliminary study. PLoS One.

[100] Yang Z, Zhang X, Fang M (2019). Preoperative diagnosis of regional lymph node metastasis of colorectal cancer with quantitative parameters from dual-energy CT. AJR Am J Roentgenol.

[101] Al-Najami I, Lahaye MJ, Beets-Tan RGH, Baatrup G (2017). Dual-energy CT can detect malignant lymph nodes in rectal cancer. Eur J Radiol.

[102] Zhou Z, Liu Y, Meng K (2019). Application of spectral CT imaging in evaluating lymph node metastasis in patients with gastric cancers: initial findings. Acta Radiol.

[103] Al-Najami I, Drue HC, Steele R, Baatrup G (2017). Dual energy CT—a possible new method to assess regression of rectal cancers after neoadjuvant treatment. J Surg Oncol.

[104] Benveniste AP, de Castro FS, Broering G (2017). Potential application of dual-energy CT in gynecologic cancer: initial experience. AJR Am J Roentgenol.

[105] Elsherif SB, Zheng S, Ganeshan D (2020). Does dual-energy CT differentiate benign and malignant ovarian tumours?. Clin Radiol.

[106] Rizzo S, Femia M, Radice D (2018). Evaluation of deep myometrial invasion in endometrial cancer patients: is dual-energy CT an option?. Radiol Med.

[107] Jiang C, Yang P, Lei J (2017). The application of iodine quantitative information obtained by dual-source dual-energy computed tomography on chemoradiotherapy effect monitoring for cervical cancer: a preliminary study. J Comput Assist Tomogr.

[108] Tawfik AM, Razek AA, Kerl JM (2014). Comparison of dual-energy CT-derived iodine content and iodine overlay of normal, inflammatory and metastatic squamous cell carcinoma cervical lymph nodes. Eur Radiol.

